# Is global health really global?

**DOI:** 10.3402/gha.v6i0.20671

**Published:** 2013-03-27

**Authors:** Peter Byass

**Affiliations:** Umeå Centre for Global Health Research, Umeå University, Sweden

This editorial is based on a keynote address given at the International Conference on Global Public Health, Colombo, Sri Lanka, in December 2012. It accompanies a set of papers which were also presented at the conference. So far, these papers describe a range of global health issues, from the health status of the United Arab Emirates (1) through to social determinants of health in India (2). Two papers from Rwanda (3) and India (4) consider specific aspects of oral public health, which was a major sub-theme of the conference.

## Where did global health come from?

An evolutionary process has occurred in thinking about how the world sees health. In the predominantly colonial era of the early 20th century, an important medical speciality known as tropical medicine emerged out of concerns about ill health associated with increasing travel to the tropics. Particularly in Europe, great institutions of tropical medicine grew in major port cities such as London, Liverpool, Antwerp and Amsterdam. An example of local relevance to Sri Lanka is a British description of a malaria epidemic in 1930s Ceylon (5).

As colonialism was superseded by independence, so the models for handling health problems needed to change; the concept of international health evolved. This included a broadening into issues of health services and delivery of care, as well as specific diseases. Nevertheless, it was still a largely northern concept, concerned with the health of people somewhere else. At the same time, at least in its better manifestations, international health involved more of the increasing number of local health professionals in the areas of interest. An example from Sri Lanka in the 1980s is joint research between the Universities of Colombo and Edinburgh on malaria immunity (6).

In the new Millennium and the 21st century, increasing globalisation in many fields left the concept of international health looking somewhat dated. Local situations were increasingly seen in their global context, for example, the importation of chikungunya and dengue fevers into Italy from, among other places, Sri Lanka (7). National and geographic boundaries may not have the defining role in health that they once had. This has partly come about as rapid technological developments have enabled communications and collaborations to happen much more effectively. It is becoming more common for health research projects to be more global than local, with many funders promoting international consortia which often include northern and southern partners (8).

However, simply regarding everything as global health may also be an unhelpful over-simplification. A few things, like the health of astronauts in the space station (9) might be unambiguously beyond the scope of global health, but in reality some areas of health and medicine clearly benefit more than others from being considered in a global context. Beaglehole and Bonita proposed a definition of global health as ‘collaborative trans-national research and action for promoting health for all’ (10).

**Figure 1:**
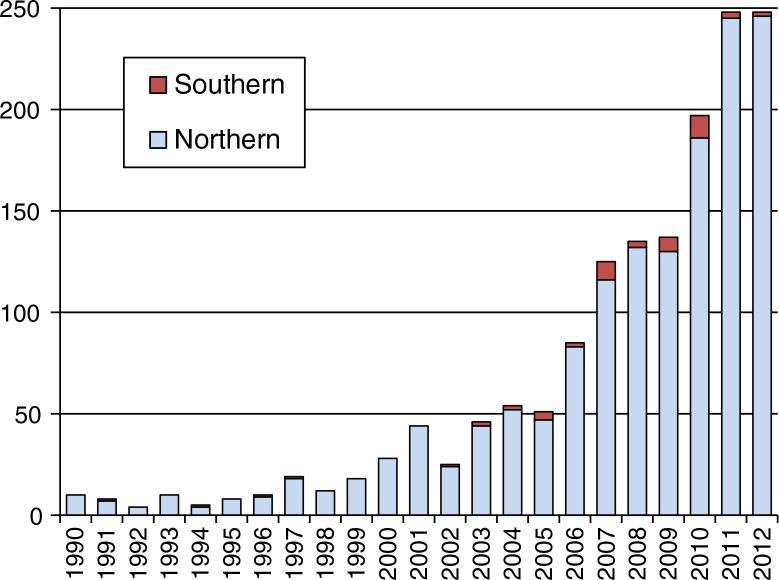
PubMed titles with ‘global health’ from southern and northern institutions.

During the past decade, global health has been widely embraced as a key concept in northern America and Europe. An approximate exponential increase in PubMed titles including ‘global health’ is evident from Fig. 1 over the past 20 years or so. However, when these titles are split between those originating from northern and southern institutions, it becomes clear that global health, despite its rapid growth, so far remains a largely northern concept. This may be a crude analysis, but the main trends are all too clear.

## What are the big issues facing global health?

Global health faces a multitude of challenges, but there are five areas that encompass many of the major issues:

- Failing to ‘make each and every person count’ [Ban Ki-moon (11)].
- Understanding the global landscape of non-communicable diseases; HIV/AIDS; changing patterns of infectious diseases and injuries.
- Recognising that threats to health have causes, and that those causes also have causes.
- Considering how global processes like climate change might affect health.
- Lack of universal free access to health information.

The world's continuing failure to count people properly is one of the emerging scandals of the 21st century. As a matter of basic human rights, every individual should have an official identity, and, of great importance in the health sector, ultimately a properly recorded cause of death. Currently poorer people are much less likely to have their deaths recorded, and this introduces a strong systematic bias into health data (12). As a consequence, a lot of health planning and policy making relies on various modelled estimates of what is actually happening, which may be the best available option, but is by no means adequate. WHO Director-General Margaret Chan has said ‘we must not forget that the real need is to close the data gaps, especially in low-income and middle-income countries, so that we no longer have to rely heavily on statistical modelling for data on disease burden’ (13). An important element in this is making appropriate tools available for vital registration procedures, such as our InterVA models for automated cause of death attribution (14).

**Figure 2:**
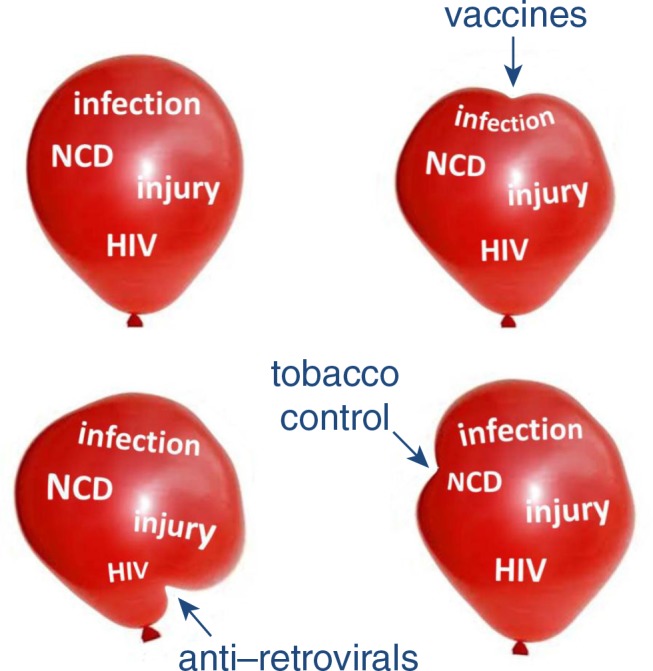
A conceptual view of overall disease burden and possible effects of specific interventions.

The global landscape of health and disease is changing rapidly and has to be considered as a dynamic entity. If the whole spectrum of morbidity and mortality is conceptualised as a balloon, then external influences such as major health interventions will lead to compression and expansion in different areas, as depicted in Fig. 2. There are also long-term trends towards greater life expectancy in most parts of the world, which should be regarded as a measure of success in terms of increasing health, but which also brings about changes in patterns of age-dependent morbidity and mortality as more people experience life at older ages. This also highlights the need for effective and current health information systems, in order to track changes in disease burden dynamically.

So-called ‘social determinants of health’ (SDH) have been increasingly recognised in recent years as an important component for understanding how patterns of health and disease manifest in populations. Sometimes referred to as ‘the causes of the causes’, SDH reflect factors that influence health but are generally unequally distributed in a society. They may themselves be driven by other up-stream factors. WHO, via its Commission on Social Determinants of Health, has called for action toward ‘closing the health gap in a generation’ (15).

The potential effects of changes in climate on human health constitute a relatively new area of concern, which operates as a top-level global factor. Part of the challenge is bringing together wisdom from a wide range of disciplines such as climatology, environmental science, and energy use, in order to understand effects on health. Nevertheless, it is now widely accepted that climate change is likely to affect human health, including effects on occupational health and migration. Thus, it is important that global health engages with climate issues in order to minimise adverse effects and maximise health and climate co-benefits (16).

Restricted access to health information on a global basis remains a major constraint for advancing global health. Although the open-access publishing model, as used by *Global Health Action*, is becoming more widespread, there is still a considerable volume of health research and information that is hidden behind frustrating pay walls, making it particularly inaccessible for researchers and policy-makers from low- and middle-income countries. There is some movement in this area, particularly as research funders are increasingly willing to pay for open-access publishing, and in some cases insisting that this be done (17).

## So is global health really global?

It seems clear that global health as a concept has not yet achieved southern ownership. This may be a transitional stage, but it also means that the future of the concept of global health is at risk: if global health does not achieve more general global ownership in the relatively near future, it may go into terminal decline as a concept. This would be unfortunate against the background of an increasingly globalised world in many non-health sectors. To ensure the future of global health, rapid progress is needed on making everyone count, documenting the dynamics of disease burdens, understanding SDH and the effects of climate on health, as well as making health information a universally accessible public good. In conclusion, global health is not yet global. Hopefully it soon will be.

*Peter Byass* Umeå Centre for Global Health Research Umeå University Sweden
